# Modern microbialites harbor an undescribed diversity of chromerid algae

**DOI:** 10.1186/s40793-026-00852-4

**Published:** 2026-01-14

**Authors:** Anthony M. Bonacolta, Patrick J. Keeling

**Affiliations:** Department of Botany, University of British Columbia, 6270 University Blvd #3156, Vancouver, BC V6T 1Z4 Canada

**Keywords:** Algae, Chromerids, Microbialites, Thrombolites, Protists, Coral reefs, Apicomplexa, Phycology

## Abstract

**Background:**

Chromerid algae are the closest photosynthetic relatives of apicomplexan parasites. While chromerids have been central to understanding the evolutionary transition from free-living algae to parasitism within Apicomplexa, their ecology remains poorly understood. Although often considered coral-associated symbionts, emerging evidence suggests this link is incidental and that chromerids may be more broadly associated with calcium carbonate environments, including microbialites. These microbial structures represent modern analogues of ancient reef-like ecosystems but are difficult to study due to their rarity and protected status as world heritage sites. Prokaryotic members of the microbialite microbiome have been studied at length, while the microeukaryotes associated with these environments have gone mostly ignored. To further investigate the link between microbialites and chromerid algae, we re-analyzed previously published microbialite sequencing data with the aim of investigating chromerid diversity and distribution.

**Results:**

Through a novel plastid-focused metagenomic binning workflow combined with re-analysis of rRNA metabarcoding data, we reveal that chromerid algae are consistent associates of microbialites across diverse marine and freshwater environments worldwide. Most notably, we report the first recovery of plastid genomes from microbialite-associated chromerids: a complete *Vitrella brassicaformis* plastid genome and a second, partial plastid genome from a previously undescribed *Chromera*-related lineage in Highborne Cay thrombolites. This partial plastid genome contained photosystem genes, confirming this novel *Chromera*-related lineage as a photosynthetic chromerid. These findings not only expand the known ecological and biogeographic range of chromerids but also provide evidence for their overlooked diversity.

**Conclusions:**

Our analyses prove that this overlooked algal lineage is not found exclusively associated with corals, but instead occurs across a wide range of microbialite habitats, including those found in freshwater. By extending their known distribution beyond coral hosts and the marine environment, our results not only highlight the diversity and ecological range of the most recently discovered algal lineage but also broaden our understanding of the ancestral lifestyles that may have preceded apicomplexan evolution. This research underscores the value of targeted mining of public sequencing datasets to address specific ecological questions, particularly in rare or hard-to-access environments such as microbialites.

**Supplementary Information:**

The online version contains supplementary material available at 10.1186/s40793-026-00852-4.

## Background

Chromerid algae and the heterotrophic colpodellids together make up the chrompodellids, which are the closest known relatives to apicomplexan parasites. The ancestor of apicomplexans and chromerids is thought to have possessed photosynthetic chloroplast much like those of chromerids, and which has been reduced to a non-photosynthetic apicoplast in apicomplexans. As apicomplexans are prolific parasites of animals, including humans, their adaptation to parasitism from algal ancestors exemplified by their plastid evolution has garnered significant interest, and chromerids were instrumental in elucidating that evolution. But the chromerids were also the first new algal group discovered in 100 years, and because so much attention has focused on comparisons with apicomplexans, the ecology of chromerid algae has remained surprisingly mysterious and unexplored. Since their discovery almost two decades ago, there are still only two described Chromerid algae, *Chromera velia* [1] and *Vitrella brassicaformis* [2]. While these two species were both initially isolated from coral in Australia, significant divergence in their morphologies, life histories, and evolution have always been apparent [2]. Nuclear gene phylogenies also support this by showing they are distantly related within the larger chrompodellid lineage: *V. brassicaformis* branches near the base of the chrompodellids with the mollusk parasite *Piridium sociable*, whereas *C. velia* branches with free-living predators *Colpodella*, *Voromonas*, and *Alphamonas* [3, 4]. Their plastid genomes are similarly divergent, with *C. velia* bearing a linear, 120 kb genome, while *V. brassicaformis* possesses a circular gene-rich 85 kb genome [5]. Their ecological roles are also seemingly divergent, but here we have much less data to go on: we know almost nothing about their role in natural environments and critical questions, especially their association with coral, are still debated. After first being presumed to be in an intracellular photosymbiotic association with coral [1, 6], the transcriptomic response of coral larvae exposed to *C. velia* was shown to more resemble parasite invasion*,* and markedly low colonization levels were observed compared to Symbiodiniaceae [7, 8]. Closer analyses of environmental data also suggested most chromerid sequences are more consistent with growth on coral surfaces, as opposed to being directly associated with coral tissue [9, 10]. Marker gene evidence of chromerid and other apicomplexan-related lineages (ARLs) in environments outside coral reefs further supports a broader ecological range for these algae [9, 11]. For instance, *Vitrella*-related 18S and plastid 16S rRNA gene sequences (classified as ARL-I), are mostly found on coral reefs, but are also present in a range of calcium carbonate-rich environments like reef sediments and even microbialites [3, 9, 11].

Microbialites are microbially-induced formations of trapped, bound, or precipitated sediment that exhibit a range of mineralogies, although calcium carbonate (CaCO_3_) is the most widespread [12]. Ancient microbialites offered some of the first evidence for carbonate-based reef communities, and modern microbalites offer fascinating insights into early evolution [13–15]. The potential association between Chromerid algae and microbialites is tantalizing given this evolutionary context and compositional similarity between coral and microbialite reef systems; however, microbialites are challenging to sample due to their rarity and the strict regulations surrounding access. To further study this link and to gather additional information on the biogeography of Chromerid algae, we employed new genomic binning approaches to publicly available shotgun metagenomic datasets and re-analyzed existing 18S rRNA gene datasets from microbialite environments, most of which were not previously reported to include chromerids. Furthermore, 16S rRNA gene metabarcoding surveys were also investigated for chromerid signal, showing more limited results, and exemplifying the utility of metagenomics and eukaryotic-specific marker gene surveys for studying the protistan associations of microbialites.

## Methods

### Data retrieval

**Fig. 1:**
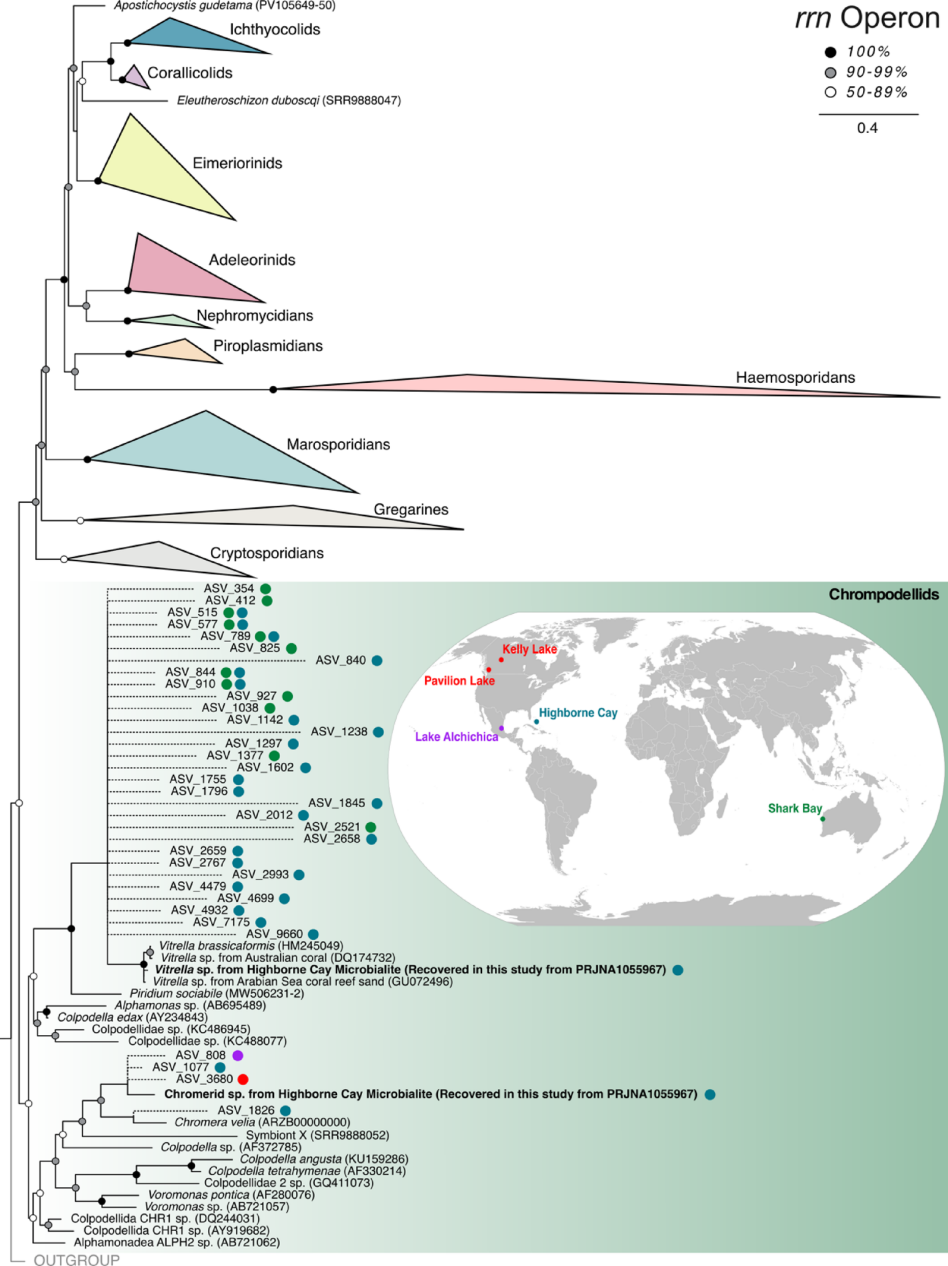
Maximum-likelihood tree of apicomplexans and chrompodellids based on the nuclear rRNA operon with EPA-placed ASVs recovered from microbialite 18S rRNA gene metabarcoding studies. World map (inset) showing locations of 18S rRNA gene metabarcoding datasets analyzed in this study. The colored dots next to each ASV represents the origin of each sequence. Note that ASV_3680 (red dot) was found in both Canadian microbialite locations

Metagenomic reads from Highborne Cay (Bahamas) thrombolites were downloaded from NCBI SRA (BioProject: PRJNA1055967), trimmed using Trimmomatic v0.39 [16], then assembled using MegaHit v1.2.9 [17]. To further assess chromerid diversity and distribution beyond this metagenomic assembly, amplicon sequence variants (ASVs) previously classified as chromerids (and confirmed by evolutionary placement analyses here) were extracted from 18S rRNA gene datasets originating from microbialites found in Pavilion Lake (Canada; freshwater), Kelly Lake (Canada; freshwater) [15], Lake Alichichica (Mexico; saline & alkaline crater lake) [18], Highborne Cay (Bahamas; saline), and Shark Bay (Australia; hypersaline; Fig. 1) [19]. These datasets encompass all publicly available 18S rRNA metabarcoding data from microbialite environments, providing the most comprehensive assessment of chromerid diversity presently possible to our knowledge. These locations are also biographically distant and range in salinity from freshwater to hypersaline, representing both geographically and chemically distinct environments. The Integrated Microbial NGS platform (IMNGS), a continuously updated platform which allows investigation of all available 16S rRNA gene amplicon studies from NCBI’s Sequence Read Archive (SRA) database [20], was also screened for ASVs 90% similar to chromerid plastid 16S rRNA genes.

### 18S rRNA gene extraction and rrn operon phylogenetics

*Vitrella-*related and *Chromera*-related 18S rRNA genes were identified and extracted from the Highborne Cay thrombolite metagenomic assembly using Blastn [21], then aligned with other chrompodellid and apicomplexan rRNA operons (18S + 28S rRNA genes) using MAFFT v7 [22]. This alignment was then trimmed using Trimal [23] (-gt 0.3 -st 0.001). A maximum-likelihood backbone tree was constructed using IQ-tree v2.1.0 with the GTR + F + R5 model (as selected by ModelFinder) [24] and 1000 bootstrap replicates. Chromerid ASVs from the 18S rRNA gene metabarcoding datasets were placed using RAxML’s evolutionary placement algorithm (EPA) [25] onto the well-supported backbone tree of the apicomplexan + chrompodellid rRNA operons to assess chromerid biodiversity across biographically distant microbialites (Fig. 1). Blastn was used to generate percent identity similarity scores for each ASV to confirmed chromerids.

### Plastid MAG binning and phylogenetics

Plastid MAGs (ptMAGs) were extracted from the Highborne Cay thrombolites metagenomic assembly using Metabat v2.18 [26] optimized for the retrieval of plastid metagenome assembled genomes (ptMAGs; -s 30000 --minContig 1500). Plastid *rrn* operon (16S + 23S rRNA genes) from both ptMAGs were extracted using barrnap [27] then used to construct a maximum-likelihood tree along with similar NCBI BLAST matches as done before for the nuclear *rrn* operons. A 500 + bp matching 16S rRNA ASV identified from the IMNGS platform search was also included in the plastid *rrn* tree. Plastid genes from the recovered ptMAGs were confirmed using hmmsearch and single-gene tree construction with FastTree. A maximum-likelihood phylogenetic tree based on 31 plastid genes was generated for the *V. brassicaformis* ptMAG. For this, single genes were first aligned with MAFFT then trimmed using trimal (-gt 0.8), before concatenation using CONCATENATOR [28]. The concatenated alignment was then used as input for IQ-tree v2.1.0 with the CPREV + I + G4 model (as selected by ModelFinder) [24] and 1000 bootstrap replicates. Plastid diagrams were generated using OGdraw v1.3.1 [29].

## Results

The full-length *Vitrella-*related 18S rRNA gene extracted from the Highborne Cay metagenomic assembly branched within a well-supported (100% bootstrap) clade with other *V. brassicaformis* sequences recovered from Australian corals and the Arabian Sea coral reef sediments and was 98.652% similar to *Vitrella brassicaformis* (Supplementary Material 1). These sequences and other environmental sequences in this clade showed minimal divergence, suggesting they all likely belong to the same species. In contrast, other *Vitrella*-related ASVs from marine microbialites all branch outside this clade and likely represent distinct lineages of *Vitrella*. The *Chromera*-related 18S rRNA gene recovered from Highborne Cay branches sister to *C. velia* but likely represents a different species or possibly even genus based on phylogenetic divergence (Fig. 1) and low similarity (92.349%) to the *Chromera velia* 18S rRNA gene (Supplementary Material 1).

**Fig. 2:**
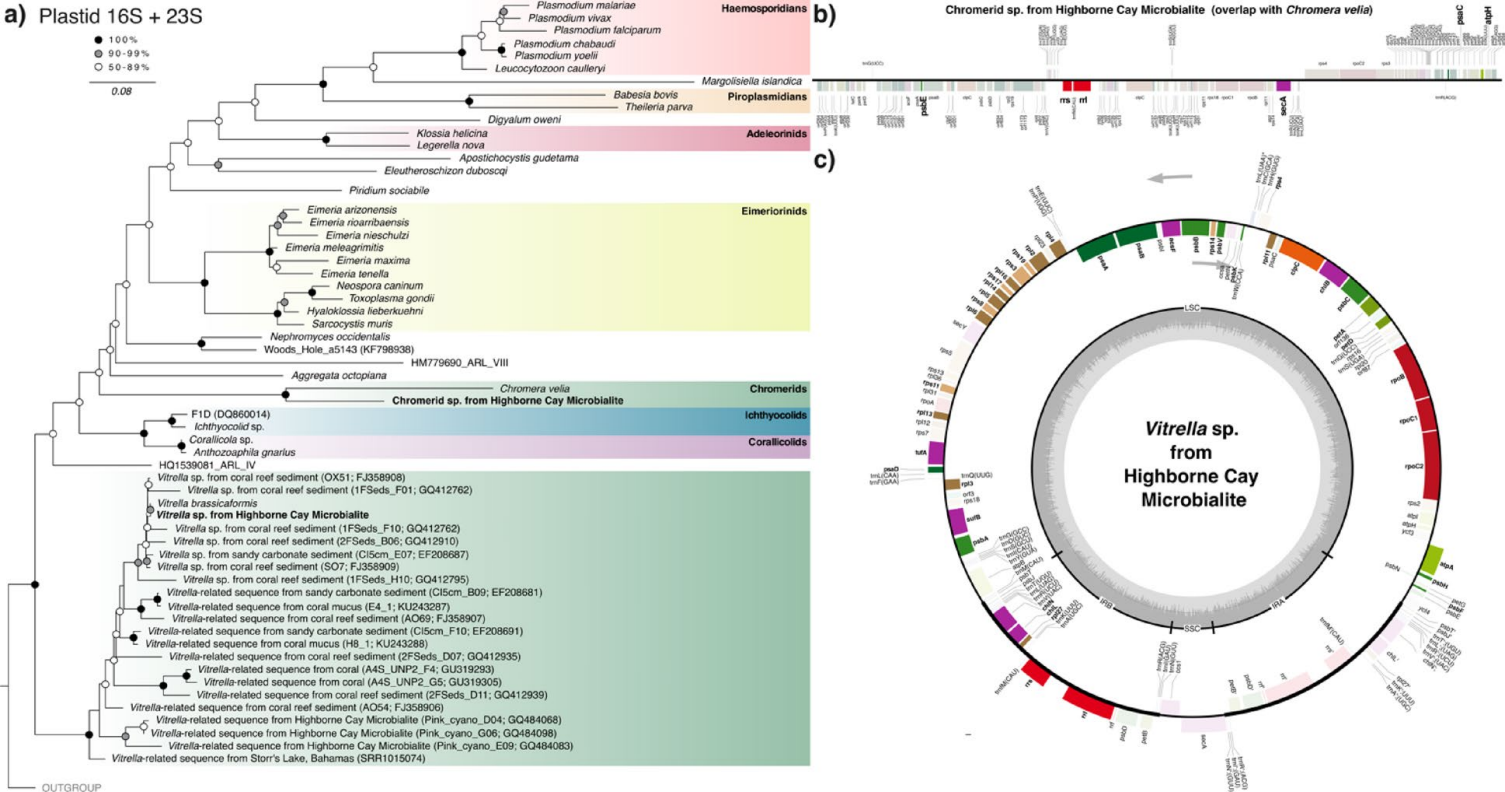
**a** Maximum-likelihood tree of apicomplexans and apicomplexan-related lineages based on the plastid *rrn* operon (16S + 23S rRNA genes). Sequences recovered here from Highborne Cay microbialites are bolded. **b** Novel *C. vela*-related plastid genome assembly recovered from a Highborne Cay microbialite MAG (metagenome assembled genome). Recovered genes (bold, fully coloured) are overlayed on the *Chromera velia* plastid genome. **c** A *V. brassicaformis*-related plastid MAG from Highborne Cay microbialites. Recovered *Vitrella brassicaformis* plastid genes (bold, fully coloured) are overlayed over the *V. brassicaformis* plastid genome

Using Metabat v2.18 [26] optimized for the retrieval of plastid metagenome assembled genomes (ptMAGs), we recovered two ptMAGs corresponding to chromerid plastids from Highborne Cay thrombolites. This binning approach allowed us to separate the closely related plastid contigs present within the metagenomic assembly into two distinct plastid genomes, further validated with phylogenetics. Extracting the plastid *rrn* operon (16S + 23S rRNA genes) from both ptMAGs revealed similar patterns to the 18S rRNA genes we retrieved, where one ptMAG *rrn* operon corresponds to *V. brassicaformis* and the other corresponds to a chromerid closely related to but distinct from *C. veli*a (Fig. 2). NCBI BLAST matches to the recovered plastid 16S rRNA genes and an IMNGS-identified sequence from Storr’s Lake (Bahamas) microbial mats clustered within the *Vitrella* lineage, exemplifying the high diversity of this group. The *Chromera*-related ptMAG was partial, at 15 kb, but nevertheless contained *psbE*, *SecA*, *psaC*, and *atpH* (Fig. 2b), the phylogenies of which confirmed each to be closely related to *C. velia* and distinct from *Vitrella*. The ptMAG corresponding to *V. brassicaformis* is ~ 82 kb and contained a majority of *V. brassicaformis* plastid genes (97.44% overall nucleotide similarity; genes were also confirmed by phylogenetic analyses to be closely related to *V. brassicaformis* homologues), however it could not be assembled into a single contig or circularized (Fig. 2c). A phylogenetic tree based on 31 plastid genes confirmed that this ptMAG is very closely related to *V. brassicaformis* (Supplementary Material 2), confirming that photosynthetic algae from this lineage live in calcium carbonate habitats beyond coral reefs.

## Discussion

We recovered a full-length *V. brassicaformis* 18S rRNA and most of its plastid genome from Highborne Cay microbialites. Gene phylogenies show that this lineage is the same as that found in coral reef environments, confirming that *V. brassicaformis* live in calcium carbonate habitats beyond coral reefs. While *Vitrella* spp. have been noted in microbialite environments previously [11], they were never observed to be particularly abundant or widely distributed. Furthermore, we found freshwater and marine chromerid ASVs from microbialites cluster with a novel chromerid lineage related to but distinct from *C. velia* (Fig. 1). Biological support for the freshwater ASV (ASV_3680) is strong considering it is found in multiple samples across two different locations [15]. Its presence amongst marine-associated microbialite ASVs further supports this clade as a likely unexplored, yet widely distributed *Chromera*-related lineage associated specifically with microbialite environments. The hypersalinity of shark bay microbialites inhibits coral reef growth [30], thus providing additional evidence for a microbialite-specific association, as opposed to contamination from nearby coral reef environments. This is the first definitive evidence of *Chromera*-related sequences recovered outside of a coral reef habitat. The presence of photosystem genes within the *Chromera*-related ptMAG also confirms this organism as photosynthetic, altogether demonstrating the presence of uncultured, photosynthetic *Chromera*-related algae in modern microbialites.

Taken together this work shows that *V. brassicaformis* and a novel, photosynthetic chromerid alga live within modern microbialites, expanding the known distribution of both subgroups of chromerid algae beyond coral reef environments. Both the current data and previous analyses of environmental ASV data show that chromerids are not normally highly abundant in most environments (with rare exceptions [31]), but their consistent presence in similar but geographically distant environments is evidence of an important ecological niche fulfilled by these algal lineages. *Ostreobium quekettii*, a coral-associated euendolithic alga [32], is also found consistently within marine microbialites [15], which may offer clues as to the ecological function or tolerances of chromerids in this environment. For example, *O. quekettii* can thrive in the variable pH, O_2_, and light environment of the coral skeleton and microbialites. Coral and microbialite-associated chromerids may exhibit similar adaptation strategies as they are also found in both environments. Furthermore, *O. qurkettii* as an euendolith also plays a major role in remodeling calcium-carbonate habitats [33], which could hint at the possibly significant role of chromerids in structuring microbialite habitats. The consistent association with CaCO_3_ substrates across aquatic environments observed for chromerids may be explained by a few factors. CaCO_3_ substrates can buffer local pH levels, reflect light, and provide for trace nutrients, ensuring an ideal environment for photosynthesis and algal growth. Additionally, the complex structure and porous nature of these substrates can be useful for avoiding predation by aquatic grazers.

## Conclusions

Twenty years of research on these algae has yielded significant insights but mostly related to the evolution of apicomplexans and their plastids. Only two species of chromerids have been cultured or described thus far, none in the last decade, and very little insight into their ecological roles in nature have been made beyond their initial discovery. We suggest that the cryptic chromerid diversity of modern microbialites should be prioritized in future culturing efforts. In culture, *C. velia* tends to form a brownish, endophytic layer on the cultivation flask [34] and the morphological form of *C. velia* is influenced by salinity [6], suggesting that the undescribed chromerids of hypersaline microbialites (i.e. Shark Bay, Australia) may be observed as non-motile occoids. From what we know about the drastic differences in morphology and genome evolution of the two cultured chromerids, a new culture of the novel *Chromera*-related algae would further expand the diversity of various biological characteristics of the group still more, and a more detailed appraisal of their ecological and physical position within the microbialite community should shed light on these characteristics of the group more broadly in complex reef communities. This research underscores the value of targeted mining of public sequencing datasets to address specific ecological questions, particularly in rare or hard-to-access environments such as microbialites.

## Supplementary Information

Below is the link to the electronic supplementary material.

Supplementary Material 1. Similarity scores of the recovered 18S rRNA Genes to Chromera velia and Vitrella brassicaformis generated using BLASTn

Supplementary Material 2. Maximum-likelihood tree of apicomplexans and apicomplexan-related lineages based on 31 plastid-encoded genes, including genes recovered from the V. brassicaformis-related plastid genome

## Data Availability

The raw data used for this project was retrieved from the NCBI SRA database. Partial plastid assemblies, SGTs, and raw tree files can be found on GitHub at: https://github.com/Abonacolta/microbialite_chromerids. Recovered 18S rRNA genes have been deposited onto NCBI GenBank under the following accession numbers: PV865597 & PV865598.

## Abbreviations

CaCO_3_: Calcium carbonate
ASV: Amplicon sequence variant
EPA: Evolutionary placement algorithm
ptMAG: Plastid metagenome assembled genome
