# Supplementary figures and images for: Modern microbialites harbor an undescribed diversity of chromerid algae
Source: Environ Microbiome. 2026 Jan 14;21:25. doi: 10.1186/s40793-026-00852-4 (PMC12888705; doi:10.1186/s40793-026-00852-4)

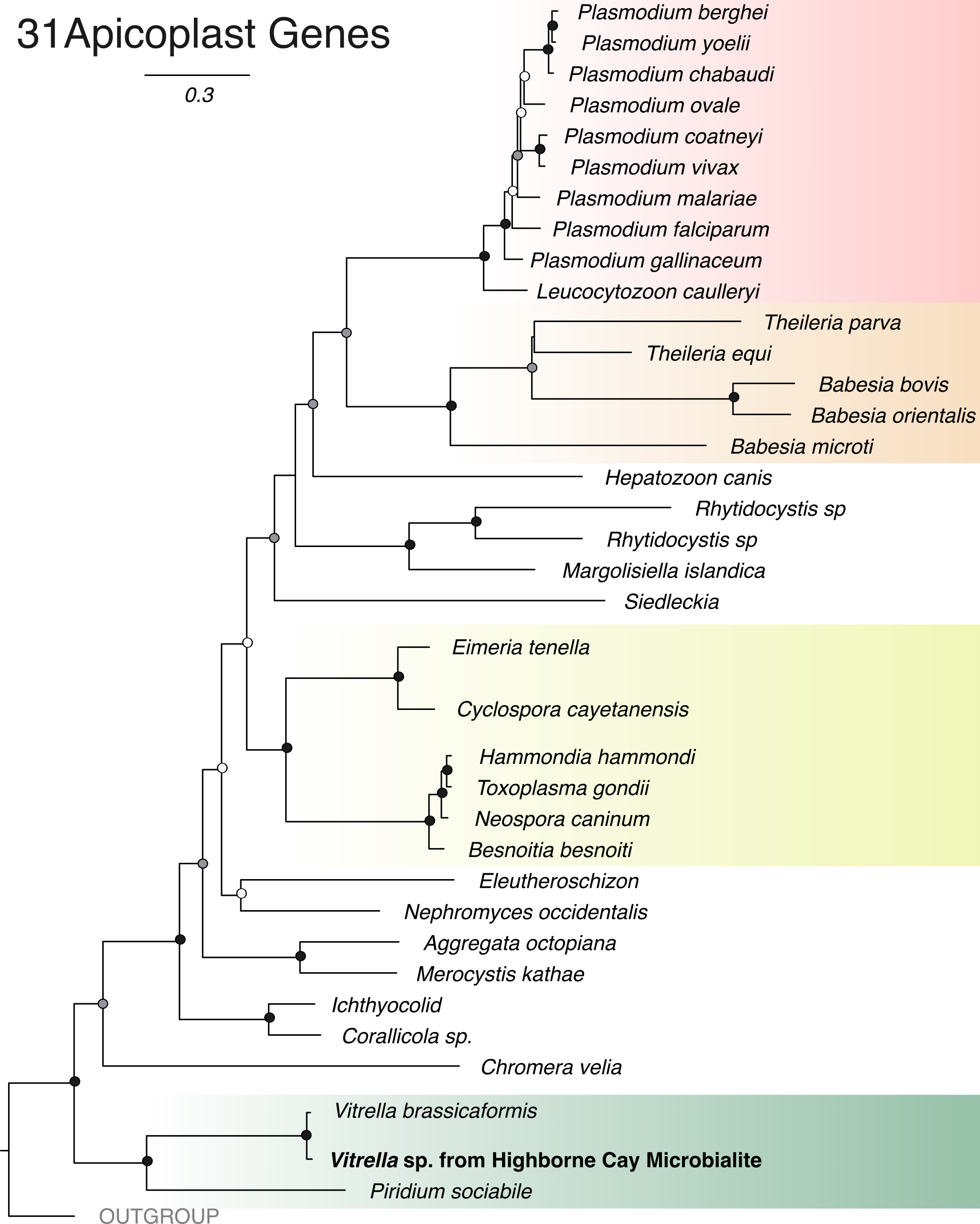

Supplement: Supplementary file 2 — Supplementary Material 2. Maximum-likelihood tree of apicomplexans and apicomplexan-related lineages based on 31 plastid-encoded genes, including genes recovered from the V. brassicaformis-related plastid genome [file 40793_2026_852_MOESM2_ESM.tiff]
